# Report for the Sixth Meeting of the International Society for Zinc Biology (ISZB-2019)

**DOI:** 10.3390/ijms21020611

**Published:** 2020-01-17

**Authors:** Toshiyuki Fukada, Taiho Kambe

**Affiliations:** 1 Molecular and Cellular Physiology, Faculty of Pharmaceutical Sciences, Tokushima Bunri University, 180 Nishihama-Boji, Yamashiro, Tokushima 770-8514, Japan; 2 Division of Integrated Life Science, Graduate School of Biostudies, Kyoto University, Kyoto 606-8502, Japan

**Keywords:** zinc, conference report, ISZB

## Abstract

The sixth meeting of the International Society for Zinc Biology (ISZB-2019) was held on September 9–13, 2019 in Kyoto, Japan. The meeting attracted 215 participants, had four plenary speakers, ten scientific symposia, two oral sessions, and one poster discussion session. In this chapter, we describe the outcomes and events of this very successful meeting.

## 1. Introduction

The International Society for Zinc Biology (ISZB) was launched in 2007 with the goal of providing a deep understanding of the roles of zinc in life [1]. Since then, it has held academic conferences every other year around the world, including Banff (Canada), 2007; Jerusalem (Israel), 2009 [2]; Melbourne (Australia), 2012; Asilomar (United States), 2014; and Pyra (Cyprus), 2017 [3]. 

The 6th conference was held in Kyoto and the academic theme was “The New Sunrise of Zinc Biology” [4]. During the preparation, we, as organizers of the ISZB-2019, had set the following three goals: 1: Updating our knowledge of zinc biology; 2: Promoting international collaboration; 3: Training young investigators. To put the goals into practice, we planned plenary lectures by four outstanding scientists, ten symposia including a session for young investigators, two oral sessions, and a poster session. We specifically focused on providing young investigators with many scientific opportunities, promoting interactions among them and with more seasoned scientists. We celebrated the ten awardees of the Metallomics Young Investigator’s Prize and the Metallomics Poster Prize aimed at honoring excellent investigations conducted by young researchers.

## 2. Meeting Overview

### 2.1. Keynote Lectures

Bulleted lists look like this: We invited four plenary lecturers who brought their fascinating research outcomes that have greatly impressed attendees. Tom O’Halloran (Northwestern University, USA) gave his talk entitled, “Zinc Fluxes in Control of Gamete Function: Mechanistic Insights into the Oocyte to Egg Transition and the Sperm-Egg Interaction.” Nickolas Tonks (Cold Spring Harbor Laboratory, USA) gave a lecture titled, “From Phosphatase-Based Therapeutics to the Discovery of a Novel Metal Chelator. Eric Skaar (Vanderbilt University, USA) addressed the intersection of nutrition and infection at the host-pathogen interface. Ananda Prasad (Wayne State University, USA) lectured about zinc deficiency, and the talk was entitled “Discovery and Impact of Zinc on Health: Biomarkers of Zinc Deficiency”. All lectures enhanced our understanding of zinc biology. 

### 2.2. Symposia and Oral Sessions

We organized ten symposia with session topics covering almost all the life science fields which stimulated attendees’ discussions. Covered topics and chairs of each symposium (SP) were as follows: SP1 (zinc sensor), M. Merkx and C. Fahrni; SP2 (neurology 1), A. Bush, E. Aizenman; SP3 (physiology and genetics), M. Hershfinkel, S. Kelleher; SP4 (young investigators), T. Fukada, T. Kambe; SP5 (immunology and inflammation), L. Rink, D. Knoell; SP6 (biochemistry), W. Maret, D. Fu; SP7 (cell biology and cancer), K. Taylor, I. Sekler; SP8 (diabetes), H. Yasui, L. Huang; SP9 (neurology 2), A. Takeda, JY. Koh; and SP10 (zinc therapy), H. Kodama, M. Moriyama. SP10 was co-organized by the Japanese Society for Zinc Nutritional Therapy (JZNT) [5] and simultaneous English-Japanese interpretation was provided for local health care workers including doctors, nurses, pharmacists and dietician, thus allowing them to fully understand the topics presented. Five young researchers were awarded the Metallomics Young Investigator’s Prize [6] and delivered their talks in the SP4 (young investigators). Most topics discussed in each session are included in this special issue.

In addition to symposia, oral session speakers selected by the organizers presented a broad range of new discoveries and approaches using various models and exploring the role of zinc homeostasis in cell physiology and molecular biology.

### 2.3. Symposia and Oral Sessions

We had 81 poster presentations, which were exhibited throughout the four days of the conference allowing attendees to read them at their convenience, sharing new data and ideas regarding the role of zinc in a large number of biological systems. Poster presentations covered a variety of aspects of zinc biology, as in the case of the symposia, including zinc in health and disease; zinc proteins, transporters, receptors, channels, and compounds; and zinc signaling. A diverse poster presentation session provided a stimulating environment for the interaction among young researchers, students, and established scientists, representing a good training opportunity for the next generation of zinc biologists. Five young researchers were awarded the Metallomics Poster Prize [6].

### 2.4. The Frederickson Prize

This year the Frederickson Prize was awarded for the first time as the official prize of the ISZB and was open to all disciplines related to zinc research. The prize is named after Christopher F. Frederickson, one of the founders of the zinc signal conferences, which finally became ISZB. In ISZB-2019, we awarded the prize to Wolfgang Maret (King’s College of London), a former president and one of the founders of the ISZB. On the final day, we also awarded Chris Frederickson an honorary ISZB membership.

Not only the scientific events, but also the social events including an excursion, helped us to extensively interact with one another. The information about all the programs is available on the meeting homepage [4].

## 3. Final Remarks

After the conclusion of the ISZB-2019, we administered a survey to learn about the impression and thoughts that the attendees had during and after the conference. The majority of the responses were very positive, especially for the use of the same venue for the conference and accommodation, which provided attendees many opportunities to interact with one another. Based on the results of the survey, we are proud to conclude that the ISZB-2019 was a very successful meeting (See group photo in the meeting home page [4]).

Finally, many thanks to all the attendees and the generous funding from academic foundations and companies who supported ISZB-2019. Without their support the conference would not have been as successful. 
